# A Two-Stage Approach for Medical Supplies Intermodal Transportation in Large-Scale Disaster Responses

**DOI:** 10.3390/ijerph111111081

**Published:** 2014-10-27

**Authors:** Junhu Ruan, Xuping Wang, Yan Shi

**Affiliations:** 1 Institute of Systems Engineering, Dalian University of Technology, Dalian 116024, China; 2 School of Electrical and Electronic Engineering, University of Adelaide, Adelaide 5005, Australia; E-Mail: junhu.ruan@adelaide.edu.au; 3 School of Business, Dalian University of Technology, Panjin 124221, China; 4 General Education Center, Tokai University, Kumamoto 862-8652, Japan; E-Mail: yshi@ktmail.tokai-u.jp

**Keywords:** large-scale disasters, medical supplies, intermodal transportation

## Abstract

We present a two-stage approach for the “helicopters and vehicles” intermodal transportation of medical supplies in large-scale disaster responses. In the first stage, a fuzzy-based method and its heuristic algorithm are developed to select the locations of temporary distribution centers (TDCs) and assign medial aid points (MAPs) to each TDC. In the second stage, an integer-programming model is developed to determine the delivery routes. Numerical experiments verified the effectiveness of the approach, and observed several findings: (i) More TDCs often increase the efficiency and utility of medical supplies; (ii) It is not definitely true that vehicles should load more and more medical supplies in emergency responses; (iii) The more contrasting the traveling speeds of helicopters and vehicles are, the more advantageous the intermodal transportation is.

## 1. Introduction

Timely relief supplies transportation takes an important role in emergency responses, directly affecting the efficiency and effectiveness of disaster relief [1,2,3]. Facing different disaster responses, decision-makers often integrate different transportation modes to deliver relief supplies as soon as possible.

In this work, we focus on a kind of disaster response situation: Due to the long distance among supply nodes and demand nodes or the cut off of key roads to affected areas, helicopters are used to transport medical supplies to temporary distribution centers (TDCs) and then vehicles at TDCs are used to transit the received medical supplies to medical aid points (MAPs).

The above intermodal transportation of helicopters and vehicles has been gradually used in emergency practices due to its specific advantages in challenging disaster situations:

- Helicopters are not subject to existing transportation networks and can fly straight to affected areas, which could sharply shorten the delivery time of medical supplies.
- Helicopters can take off and land vertically at relatively small places. Thus, it is flexible and quick to select and clear up places as TDCs for receiving medical supplies from helicopters.
- In destructive disasters such as earthquakes and floods, the cut off of key roads often makes helicopters the most effective transportation mode to isolated affected areas.

However, due to the limited number of helicopters, it is difficult or impossible to transport medical supplies to every MAP by helicopters in large-scale disaster responses. In general, TDCs are set up for receiving badly needed medical supplies from helicopters and then transiting the supplies to each MAP by vehicles. Since helicopters could take off and land vertically at relatively small places, in the work we assume that any place in affected areas could be located as TDCs.

**Figure 1:**
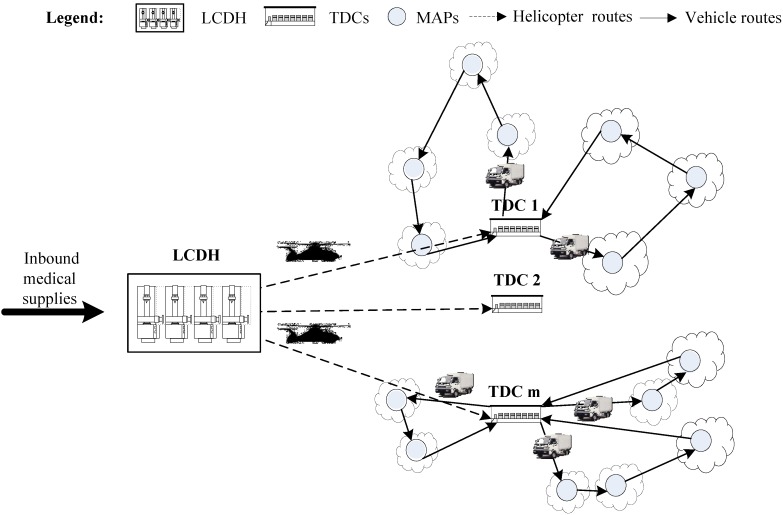
A simplified diagram of the intermodal transportation.

Figure 1 illustrates a simplified diagram of our focused problem. In response to a large-scale disaster, all inbound medical supplies are first collected at a large collecting and distributing hub (LCDH), then transported to TDCs by helicopters and finally delivered to corresponding MAPs by vehicles. The locations of LCDH and MAPs are given. The locations of TDCs are unknown and need to be determined.

There are two subproblems that need to be solved: one is where TDCs should be located, and the other is how to arrange the delivery routes. In this work, we develop a two-stage approach for the above intermodal transportation problem. **Stage I:** A fuzzy-based method is presented to determine the locations of TDCs and assign MAPs to each TDC. In this stage, the intermodal transportation network is constructed. **Stage II:** Based on the constructed intermodal transportation network, an integer-programming model is built to produce the delivery routes. Our proposed approach has potential applications in some challenging emergency situations such as vaccine delivery in large-scale infectious diseases, blood transportation after earthquakes, and antidote delivery in chemical and biological attacks.

The remainder of this paper is organized as follows. In Section 2, we give a brief review on related works. In Section 3, we present our approach, including the two-stage problem formulation, a fuzzy-based method for selecting TDCs and assigning MAPs in Stage I, and an integer-programming model for determining the delivery routes in Stage II. In Section 4, we conduct numerical experiments to verify the effectiveness of the two-stage approach. Conclusions are drawn in Section 5, with some recommendations on future studies.

## 2. Literature Review

Many studies have been reported to achieve solutions for emergency logistics problems [4,5,6]. In the work, our decision-making problem involves: how to locate TDCs, how to assign MAPs and how to arrange delivery routes. Thus, we briefly review part of related works on the location problem and multi-modal transportation problem in emergency logistics.

Jia *et al.* built a facility location model of medical supplies and developed three heuristic algorithms to solve the model [7]. Mete and Zabinsky considered possible disaster types and magnitudes to develop a stochastic programming model, which could be used to select the storage locations of medical supplies [8]. An *et al.* considered service disruptions to propose an emergency facility location model that could determine the pre-emergency facility location planning [9]. Yarmand *et al.* focused on the vaccine allocation in infectious diseases and proposed a two-phase vaccination policy [10]. These studies have developed effective approaches for determining the location of emergency facilities and supplies, which are mainly regarding the disaster preparedness stage. In our work, the locating of TDCs depends on the distribution of MAPs in disaster responses, and the integrated decision making of locating TDCs and assigning MAPs needs to be considered. Meanwhile, most extant studies formulated crisp location models using 0–1 variables, which cannot fully reflect the closeness degrees of the MAPs assigned to the same TDC. If two MAPs are assigned to the same TDC, both their variable values in extant crisp models would be one. In fact, the distances of the two MAPs to the TDC may be different. Motivated by these observations, we will present a fuzzy-based method for locating TDCs and assigning MAPs, where the membership degree in fuzzy set theory is used to represent the closeness degree.

A few researchers have studied the multi-modal transportation of relief supplies in disaster responses. Haghani and Oh formulated the logistical problem in disaster relief management as a multi-commodity, multi-modal network flow model with time windows, and presented two heuristic algorithms to solve the model [11]. Barbarosolu and Arda formulated another multi-commodity and multi-modal network flow model for relief supplies transportation in disaster responses [12], and Özdamar modeled the emergency logistics as a multi-period multi-commodity network flow problem with different transportation modes [13]. Hu built an integer-linear-programming model for the container multimodal path selection in the context of emergency relief [14]. Najafi *et al.* proposed a multi-mode stochastic model to manage the logistics of both commodities and injured people in the earthquake response [15], and then developed a dynamic model for the same problem [16]. In these studies, multiple transportation modes including air, railway and road were simultaneously considered, aiming at selecting suitable modes with different transportation efficiencies for kinds of relief supplies in different urgency degrees. Different from the extant multi-mode emergency transportation model, we focus on a kind of intermodal transportation in disaster response situations where helicopters and vehicles are used collaboratively.

## 3. The Proposed Approach

### 3.1. A Two-Stage Problem Formulation

As Figure 1 shows, our focused problem can be described as follows. In response to a large-scale disaster, there are medical aid points (MAPs) providing medical service to their covered affected areas. These MAPs need medical supplies in different amounts due to different disaster levels and social situations in their covered affected areas. A large collecting and distributing hub (LCDH) is set up for collecting and transporting medical supplies to temporary distribution centers (TDCs) by helicopters. TDCs need to be located for receiving medical supplies from helicopters and transiting the received supplies to their covered MAPs by medical vehicles.

In disaster responses, medical supplies should be transported to MAPs as soon as possible [17,18,19]. Thus, the decision-making objective is to minimize the total duration time of all the intermodal routes. The decision subproblems include: how to locate TDCs and assign MAPs, and how to arrange delivery routes, so in this work we present a two-stage approach:

**Stage I**: In this stage, we select the locations of TDCs and determine which MAPs should be assigned to each TDC, aiming at minimizing the total distance among TDCs and their covered MAPs. This stage could determine the intermodal transportation network.

**Stage II**: Based on the located TDCs and assigned MAPs in Stage I, we develop an integer-programming model to arrange the delivery routes in the intermodal transportation network, aiming at minimizing the total duration time of all intermodal routes.

For simplicity, our developed approach is subject to the following assumptions:

(1) All inbound medical supplies are first collected at the LCDH, where the supplies are transported to TDCs by helicopters and then from TDCs to MAPs by vehicles. In emergency practices, external relief supplies are often collected at large and safe hubs [20,21].

(2) The capacities of helicopters and selected TDCs are large enough, compared to the small volume of limited medical supplies in disaster responses. In pressing times, helicopters are often unable to deliver scarce medical supplies with full capacity [22].

(3) Any place in affected areas could be located as TDCs, and the selected TDCs should cover all the MAPs. This assumption is consistent with the fact helicopters could take off and land vertically at relatively small places, and the fairness criterion widely considered in practical disaster responses.

(4) There are limited medical vehicles at TDCs for transferring medical supplies to MAPs, and the vehicles are with the same maximum capacity. Unlike conventional supplies such as food and water, medical supplies transportation need special vehicles, which can keep the supplies unspoiled and uninfected, so the number of these special vehicles is often limited in large-scale disaster responses [8].

(5) In a given delivery interval, each MAP is visited only once, and every vehicle leaves from and returns to its TDC. Emergency response is often a lasting process [13,20], so vehicles should be arranged with no repeated delivery for one MAP in a given interval. After finishing a delivery task, vehicles should return to their TDCs for the next delivery task.

(6) The distances from the LCDH to TDCs and helicopter traveling speed are known. Each helicopter serves one TDC. The distances among TDCs and MAPs, and the vehicle traveling speed are also known.

Notations used in the work are defined as follows:

*m*: The number of TDCs needed to be selected;

*C_i_*: The coordinate vector of the *i*th TDC, that is, 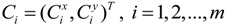;

*n*: The number of MAPs in the disaster response;

*A_j_*: The coordinate vector of the *j*th MAP, that is, 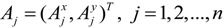;

*R_j_*: The allocated quantity of medical supplies for the *j*th MAP;

*H*: The number of available helicopters at the LCDH;

*N^C_i_^*: The set of covered MAPs of the *i*th TDC;

*n^C_i_^*: The number of the elements in *N^C_i_^*;


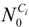
: The union of *N^C_i_^* and the *i*th TDC, whose index in the set is 0;


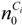
: The number of the elements in 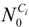, which is equal to *n^C_i_^* + 1;

*K^C_i_^*: The set of available vehicles at the *i*th TDC;

*k^C_i_^*: The number of the elements in *K^C_i_^*;

*Q_v_*: The maximum capacity of each delivery vehicle at TDCs;


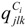
: The available load of vehicle *k* when the vehicle travels from the *j*th MAP (or the *i*th TDC) to the *l*th MAP (or the *i*th TDC), 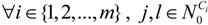;

*d_LC_i__*: The distance between the LCDH and the *i*th TDC, *i* = 1,2,…,*m*;


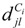
: The distance among the *i*th TDC and its covered MAPs, 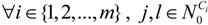;

*V_h_*: The average helicopter travel speed;

*V_v_*: The average vehicle travel speed;


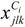
: A binary variable: 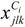 = 1 means vehicle *k* travels from the *j*th MAP (or the *i*th TDC) to the *l*th MAP (or the *i*th TDC), 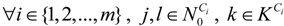; otherwise 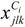 = 0;


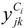
: A binary variable: 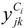 = 1 means that the *j*th MAP (or the *i*th TDC) is visited by vehicle *k*, 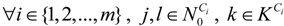; otherwise, 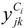 = 0.

### 3.2. Stage I: Selecting TDCs and Assigning MAPs

In this section, we propose a fuzzy-based method for selecting TDCs and assigning MAPs according to the distribution of MAPs.

In response to a large-scale disaster, the locations of *n* MAPs are denoted by *A*_1_, *A*_2_, …, *A*_*n*_, and *m* TDCs whose locations are denoted by *C*_1_, *C*_2_, …, *C*_*m*_ need to be selected. In order to minimize the total distance among TDCs and their covered MAPs, the decision criterion is to assign each MAP to its closest TDCs.

As discussed in Section 2, the binary variable in crisp location models cannot fully reflect the closeness degrees of the MAPs assigned to the same TDC. Motivated by the fuzzy theory, we use the membership degree to represent the closeness degree. Let *u_ij_* denote the membership degree of the *j*th MAP belonging to the *i*th TDC, 0 ≤ *u_ij_* ≤ 1. In order to keep the consistency with crisp binary variable, we assume that the sum of one MAP’s membership degrees to all TDCs is equal to 1, that is, *u_ij_* satisfies:

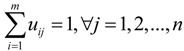
(1)


As stated above, the objective of Stage I is to minimize the total distance among TDCs and their covered MAPs. Since the membership degree, rather than a binary variable, is used to represent the closeness degree, we use the quadratic sum of the weighted distances among MAPs and TDCs to formulate the objective function of Stage I:

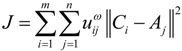
(2)
where 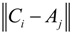 represents the distance between *C_i_* and *A_j_*, that is, 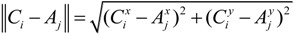, and *ω* ∊ (1,∞) is a weighted coefficient. As we can see, if 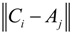 is smaller, a bigger weight 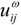 will be assigned to it, so the solutions of Equation (2) (that is, *C_i_* s) could produce the shortest total distance among TDCs and their covered MAPs.

Since the constraint of the objective Equation (2) is 
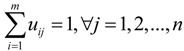
, according to the Lagrange algorithm [23,24], the constrained objective Equation (2) could be transformed into the following unconstrained objective Equation:

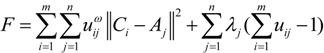
(3)
where *λ_j_* represents the Lagrange multiplier of the constraint condition (1), *j* = 1,2,…,*n*.

Taking the derivative of Equation (3), we can get the necessary conditions of minimizing the unconstrained objective function:

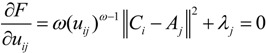
(4)

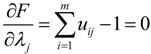
(5)

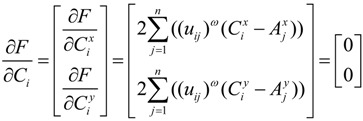
(6)


By Equations (4)–(6), we can get:

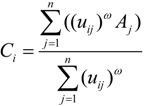
(7)

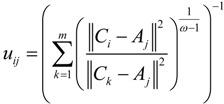
(8)


The detailed derivations of Equations (7) and (8) are respectively given in Appendix I and Appendix II. As seen from Equations (7) and (8), the location of the *i*th TDC could be determined if the locations of MAPs and corresponding membership degrees are known, and we can use iteration algorithms to get the solutions of the unconstrained objective Equation (3), that is, *C_i_* and *u_ij_*.

The termination criterion of iteration algorithms could be set as 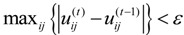, where *ε* is a given threshold between 0 and 1, and *t* represents the iteration step. After getting the *u_ij_*, we could assign the *j*th MAP to the TDC with the maximal *u_ij_* = 1,2,…,*m*.

In the following, we develop a heuristic algorithm to get *C_i_* and *u_ij_*:

**Inputs:** *m*: the number of TDCs needed to be selected; *n*: the number of MAPs; *A_j_*, *j* =1,2,…,*n*: the location of MAPs; *ω*: the fuzzy weighted coefficient of (2); *ε*: the termination threshold of the iteration.

**Outputs:** 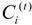: the location of selected TDCs; *U*^(*t*)^ = [*u_ij_*]_*m×n*_: the final membership degree matrix; *J*^(*t*)^: the value of the objective function (2); *N*^*C*_*i*_(*t*)^: The set of covered MAPs of TDC 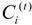.

Then, the detailed steps of the heuristic algorithm are as follows:

**Step 1:** Initialize *m*, *n*, *A_j_*, *ω*, *ε*;

**Step 2:** Randomly generate an initial matrix of membership degrees *U*^(0)^ = [*u_ij_*]_*m×n*_ such that 0 ≤ *u_ij_* ≤ 1 and 
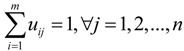
 (In details, we randomly generate the initial locations of *m* TDCs, and then use the normalized reciprocals of the distances among MAPs and the initial TDCs to determine the initial membership degrees);

**Step 3:** Use 
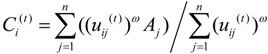
 where *t* represents the iteration step to calculate thelocations of TDCs (*i.e.*, 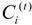, *i* = 1,2,…,*m*);

**Step 4:** Use 
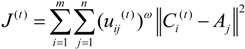
 to get the value of the objective function; if 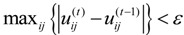, then stop the iteration and turn to Step 6 with the values of 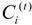, *U*^(*t*)^ and *J*^(*t*)^;

**Step 5:** Calculate the new *U* ^(*t*+1)^ using 
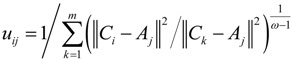
, then *t* = *t* + 1 and go to Step 3;

**Step 6:** Based on *U*^(*t*)^, calculate the maximal membership of each *A_j_* belonging to 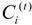, and judge the covered *A_j_* s of each 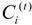 (i.e., *N*^*C*_*i*_(*t*)^); output 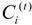, *U*^(*t*)^, *J*^(*t*)^ and *N*^*C*_*i*_(*t*)^.

### 3.3. Stage II: Arranging Delivery Routes

After selecting the locations of TDCs and assigning MAPs to each TDC in Stage I, the intermodal transportation network could be determined. Then, we need to plan the delivery routes in Stage II. In the work, we assume that each TDC is served by one helicopter in one delivery interval, so the helicopter routes are determined after we select the locations of TDCs. In this section, we develop an integer-programming model to arrange the vehicle delivery routes from TDCs to their covered MAPs.

As mentioned in Section 3.1, the objective is to minimize the total duration time of all intermodal routes. Because the volumes of medical supplies are often small and the allocated medical supplies to each TDC are often limited, the loading and uploading time at TDCs is often so short that it could be ignored. Thus, in this work we mainly consider helicopter travel time and vehicle travel time. Then, the helicopter arrival time at one TDC is the starting time of all the used vehicles at the TDC, so the duration time of each intermodal route should be equal to the sum of helicopter travel time and vehicle travel time. Thus, both helicopter travel time and the number of used vehicles at TDCs have impact on the total duration time of all intermodal routes.

**Figure 2:**
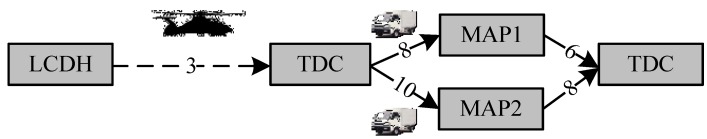
The duration time of intermodal routes.

Figure 2 gives a simple example where the numbers represent helicopter and vehicle travel time. Let us set helicopter starting time as 0. The helicopter travel time is 3, so the two vehicles have to leave from the TDC at 3. The duration times of the two intermodal routes are respectively equal to 17 (3 + 8 + 6) and 21 (3 + 10 + 8). The total duration time considered in the work is equal to 38 (17 + 21), which could be divided into two parts: helicopter travel time multiplied by the number of used vehicles (3 × 2) and vehicle travel time (8 + 6 + 10 + 8).

Thus, the optimization objective of arranging delivery routes, that is, to minimize the total duration time of all intermodal routes, is formulated as:

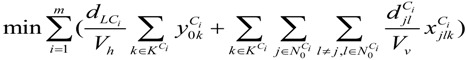
(9)


In the above formulation, 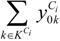 denotes the number of used vehicles starting from the *i*th TDC, which is also equal to the number of vehicle routes from the *i*th TDC, so 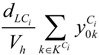 denotes helicopter travel time considered in the intermodal duration time for MAPs covered by the *i*th TDC; 
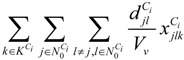
 denotes vehicle travel time considered in the intermodal duration time for MAPs covered by the *i*th TDC.

The constraints of the optimization objective Equation (9) are as follows:

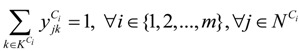
(10)

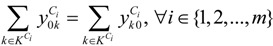
(11)

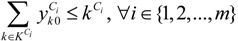
(12)

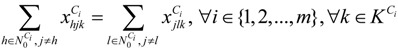
(13)

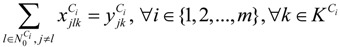
(14)

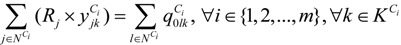
(15)

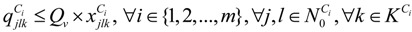
(16)

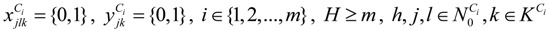
(17)


Constraint Equation (10) ensures each MAP is served only once by one of the vehicles in the delivery interval, where 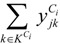 represents the number of vehicles visiting the *j*th MAP.

Constraint Equation (11) ensures the number of vehicles leaving from the *i*th TDC (that is, 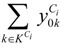) is equal to the number of vehicles returning to the *i*th TDC (that is, 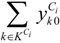).

Constraint Equation (12) guarantees the number of used vehicles does not exceed the number of available vehicles at each TDC.

Constraint Equations (13) and (14) ensure the vehicle which arrives at one MAP should leave from the MAP, where 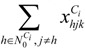 represents the number of vehicles arriving at the *j*th MAP and 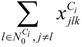 represents the number of vehicles leaving from the *j*th MAP.

Constraint Equation (15) guarantees any vehicle loads the equivalent quantity of medical supplies allocated to MAPs served by the vehicle, where 
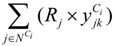
 represents the total quantity of medical supplies allocated to MAPs served by vehicle *k* and 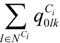 represents the total load of vehicle *k* when leaving from the *i*th TDC.

Constraint Equation (16) guarantees that the available load of vehicle *k* between the *j*th MAP (or the *i*th TDC) and the *l*th MAP (or the *i*th TDC) does not exceed the maximum capacity of vehicle *k* if traveling from the former to the later. Constraint Equations (15) and (16) together guarantee that the overall available capacity of a given vehicle does not exceed its maximum capacity.

Constraint Equation (17) defines the ranges of variables.

Meanwhile, we also formulate two performance metrics for the intermodal transportation.

(1) Average arrival time (AAT)

The optimization objective (9) mainly reflects the total duration time, but it does not specifically consider the utility of medical supplies. The utility of received relief supplies is different for each MAP if the arrival time is different [18,19,25]. The earlier the arrival time at one MAP is, the higher the utility for the MAP is. Then, in order to increase the utility of all medical supplies, the average arrival time (AAT) for all the MAPs should be shortened as much as possible, which is formulated as follows:

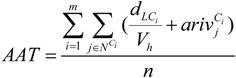
(18)
where 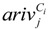 represents the elapsed time from the *i*th TDC to the *j*th MAP. Note that the vehicle return time at TDCs is not included.

(2) Biggest traveling time (BTT)

In emergency practices, the deadline policy is usually adopted, that is, the needed supplies must be transported to affected areas in some regulated period (e.g., 24 h) after the occurrence of disasters. Motivated by this observation, we formulate the biggest traveling time as one performance metric of the intermodal transportation:

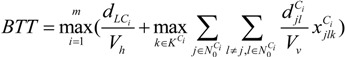
(19)


As we can see, if only in terms of BTT, the shorter it is, the better the transportation performance is.

## 4. Numerical Experiments

In this section, we conduct numerical experiments to verify the effectiveness of the developed approach, and observe several insights for real-world emergency responses.

### 4.1. Data Generation

**Figure 3:**
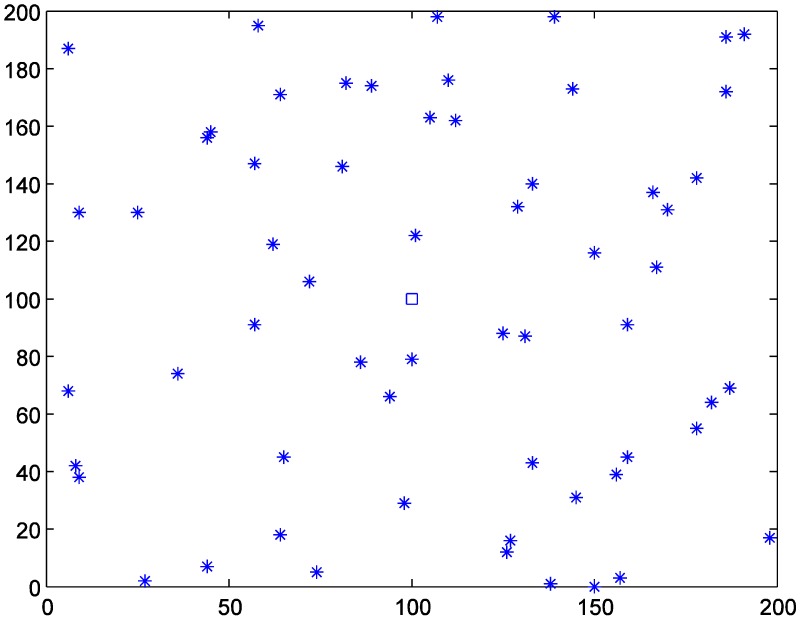
Locations of MAPs (labeled by asterisks) and the LCDH (labeled by the square).

Assume that some area is infected by an infectious disease, and sixty hospitals are set as medical aid points (MAPs) for providing medical service to victims. Considering the generality, we randomly generate the coordinates of the sixty MAPs from 0 to 200, as Figure 3 and Table 1 show. A batch of vaccines is received at the large collecting and distributing hub (LCDH) whose coordinate is set as (100, 100) and should be transported to the sixty MAPs as soon as possible. The allocated vaccines for these MAPs are as the *R_j_* columns in Table 1 show.

**Table 1:** The original data.

MAPs	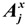	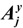	*R_j_*	MAPs	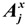	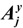	*R_j_*
**1**	139	198	1136	**31**	44	156	880
**2**	57	91	1011	**32**	156	39	722
**3**	9	130	719	**33**	129	132	632
**4**	126	12	1026	**34**	170	131	737
**5**	144	173	721	**35**	94	66	1013
**6**	101	122	1055	**36**	167	111	880
**7**	157	3	1014	**37**	125	88	765
**8**	25	130	828	**38**	182	64	786
**9**	186	191	1039	**39**	6	187	495
**10**	72	106	974	**40**	110	176	753
**11**	112	162	654	**41**	100	79	832
**12**	159	45	831	**42**	44	7	1148
**13**	36	74	616	**43**	159	91	766
**14**	198	17	672	**44**	138	1	776
**15**	191	192	870	**45**	58	195	941
**16**	62	119	667	**46**	178	55	854
**17**	65	45	798	**47**	86	78	583
**18**	105	163	794	**48**	74	5	1129
**19**	131	87	886	**49**	8	42	1021
**20**	150	0	822	**50**	6	68	907
**21**	89	174	583	**51**	133	140	723
**22**	150	116	841	**52**	178	142	850
**23**	57	147	787	**53**	186	172	803
**24**	166	137	676	**54**	187	69	775
**25**	82	175	807	**55**	45	158	817
**26**	107	198	1119	**56**	98	29	926
**27**	9	38	808	**57**	133	43	824
**28**	64	18	882	**58**	145	31	1107
**29**	127	16	599	**59**	81	146	816
**30**	27	2	790	**60**	64	171	712

### 4.2. Results of Selected TDCs

Using the proposed method for selecting TDCs and assigning MAPs in Section 3.2, we implemented the heuristic algorithm in Matlab R2012a. The related parameters of the algorithm are as follows: *ω* = 2, *ε* = 1 × 10^-5^, and the maximum number of iterations is set as 100.

**Figure 4:**
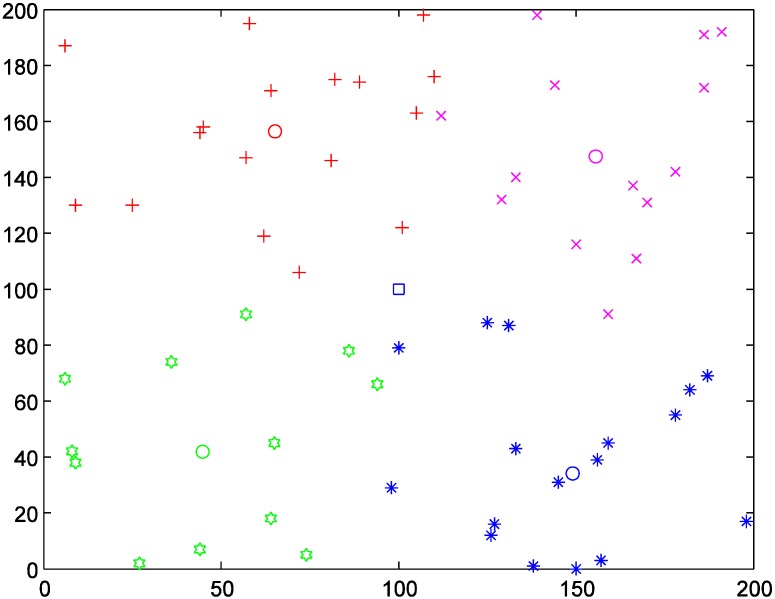
The selected TDCs and assigned MAPs with four TDCs.

To show the details, we present the results when the number of TDCs is set as four. After 43 iterations, the algorithm reached its termination criterion, with the objective value (2) being 62,411.0128. The detailed results are as shown in Figure 4 where the four circles represent the locations of selected TDCs. The assigned MAPs for each TDC are grouped in different shapes and colors.

**Figure 5:**
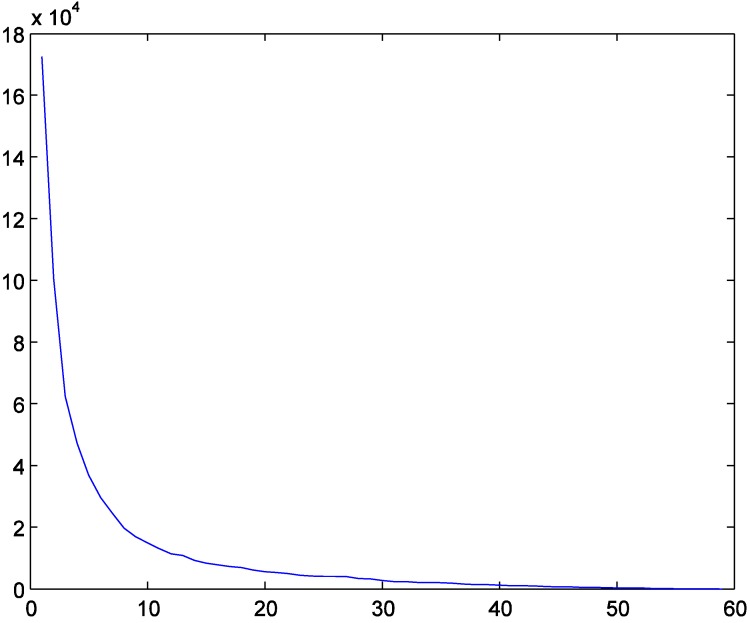
The value of objective Equation (2) with different numbers of TDCs.

Similarly, we obtained the detailed results with the number of TDCs ranging from two to 15, as Table 2 shows (For the limitation of space, the results of assigned MAPs are not given). From the results in Table 2, we can get the following observations:

(1) As the number of TDCs increases from two to 15, the value of objective function (2) definitely decreases. This means the sum of distances among TDCs and MAPs will be shortened with the increase in the number of TDCs.

(2) The decrease of the value of objective function (2) is diminishing as more TDCs are selected. In particularly, when the number of TDCs increases to 60 (the number of MAPs), the sum of distances among TDCs and MAPs will decrease to 0, as Figure 5 shows.

(3) The number of termination iterations is not in obvious relevance to the number of TDCs. All the termination iterations are within 100 (that is, the maximum number of iterations), and the longest running time of all the iterations is 5.61 Sec.

**Table 2:** Results with different numbers of the selected TDCs.

The Number of TDCs	The Selected Locations of TDCs	Iterations	The Value of Objective Equation (2)
2	(115.1215, 44.9284) (99.7824, 150.9591)	41	172,532.3624
3	(116.3484, 161.1088) (47.9796, 73.6059) (147.7658, 41.5159)	91	100,417.3994
4	(155.5038, 147.4673) (65.1837, 156.4479) (149.0295, 34.1258) (44.7962, 41.9201)	43	62,411.0128
5	(44.0622, 34.2492) (147.8524, 29.6506) (48.0010, 144.1700) (161.7073, 130.1246) (108.2454, 169.5621)	80	47,221.8533
6	(96.5021, 78.7748) (45.4646, 151.4802) (167.2060, 139.0513) (150.3087, 27.2637) (107.2651, 170.9193) (34.6524, 28.9273)	62	36,831.0511
7	(169.8822, 150.8263) (173.5107, 60.7168) (90.2298, 79.5935) (103.9641, 170.3261) (137.6201, 14.8587) (44.7909, 152.3732) (27.7608, 31.9316)	78	29,522.1085
8	(181.6705, 181.8142) (62.4966, 15.5558) (101.0444, 170.4035) (149.7739, 24.8483) (159.1980, 116.1517) (13.5109, 51.6327) (95.1028, 79.5795) (45.3501, 153.3287)	57	24,550.5521
9	(99.8256, 171.1113) (59.2840, 14.6074) (91.8373, 80.4970) (12.5390, 51.5439) (139.9251, 12.9395) (182.7777, 184.1032) (158.5230, 126.3868) (44.9873, 153.6155) (175.5131, 57.1305)	53	19,631.8894
10	(119.6735, 85.2224) (65.3041, 106.4243) (45.7608, 157.6658) (139.8517, 11.9312) (60.4220, 14.8274) (101.9682, 171.8361) (183.9530, 185.0312) (162.9210, 129.8682) (176.7298, 56.7747) (11.2449, 47.9950)	100	16,907.0872
11	(180.6445, 62.8425) (139.6802, 6.7262) (184.9137, 185.0973) (58.2463, 13.7295) (11.4684, 49.2855) (162.2845, 128.1690) (149.7515, 39.3025) (91.3246, 78.8456) (110.2706, 167.3838) (74.2499, 172.9312) (41.1820, 149.2225)	73	14,917.4491
12	(98.5393, 173.5782) (10.3469, 47.0838) (185.3382, 186.1472) (181.1156, 62.5890) (140.3170, 6.1505) (94.2870, 74.8852) (150.4305, 38.7289) (167.5805, 131.8681) (58.1328, 12.9601) (62.8250, 111.7601) (45.0970, 157.4632) (129.7547, 135.5699)	79	12,977.8993
13	(110.8082, 168.4445) (10.0571, 46.2019) (63.9118, 110.4021) (151.0760, 37.9474) (78.9164, 174.7529) (57.8343, 12.5276) (42.5859, 154.6934) (181.7441, 62.2657) (185.7684, 186.0645) (166.5562, 132.7123) (91.5287, 73.6481) (129.3355, 89.5669) (140.8238, 5.6172)	58	11,382.4811
14	(63.7141, 110.9293) (186.1755, 186.7686) (178.7301, 57.9741) (149.8187, 116.4298) (98.2565, 28.2879) (9.6585, 46.8682) (42.7754, 154.8233) (109.9915, 169.2300) (128.6312, 87.3772) (144.2500, 7.5919) (168.9176, 136.0067) (45.7029, 8.7246) (78.1378, 174.8745) (91.8098, 74.1798)	73	10,761.5505
15	(9.2761, 45.3432) (130.2494, 86.9024) (66.8312, 107.4951) (131.2589, 135.4988) (46.9954, 157.3454) (169.0423, 133.2947) (36.6269, 5.7054) (93.1979, 72.9595) (110.1389, 170.3292) (70.9212, 15.7676) (141.7105, 9.3871) (82.2789, 175.1017) (186.5786, 186.7270) (17.4716, 131.1668) (178.4745, 57.7653)	57	9216.5779

In the following section, we will generate different transportation routes with different numbers of TDCs, and analyze the performance of the intermodal transportation with these selected TDCs.

### 4.3. Results of Delivery Routes

After selecting TDCs and assigning MAPs in Section 4.2, we use CPLEX 9.0 (with default parameters and maximal running time being 3600s) to solve the optimization model in Section 3.3, which could produce the delivery routes among TDCs and MAPs. Without loss of generality, we set the average vehicle travel speed *V_v_* as 1.

#### 4.3.1. Results with Different Numbers of TDCs

In order to observe the impact of the number of TDCs on the performance of the intermodal transportation, in this subsection we set the maximum capacity of each delivery vehicle *Q_v_* as 5000 and helicopter travel speed *V_h_* as 10.

**Figure 6:**
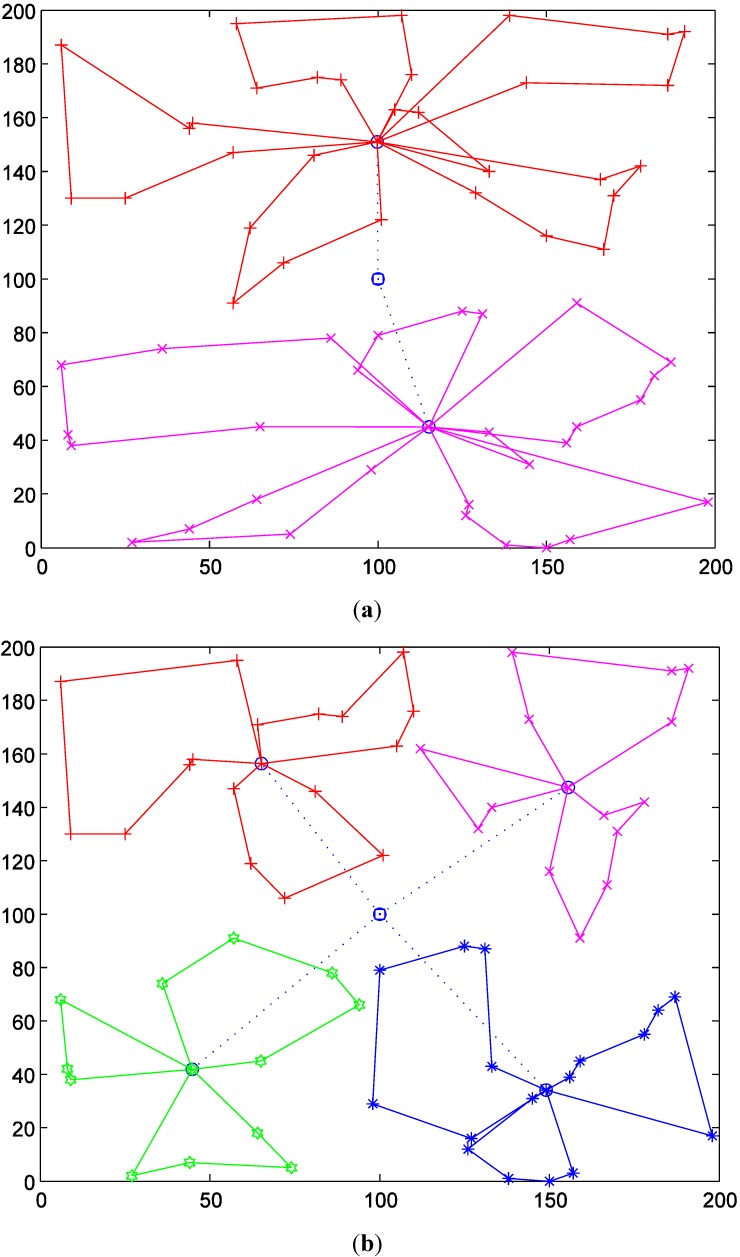
Intermodal transportation routes with two and four TDCs. (**a**) *m* = 2; (**b**) *m* = 4.

**Figure 7:**
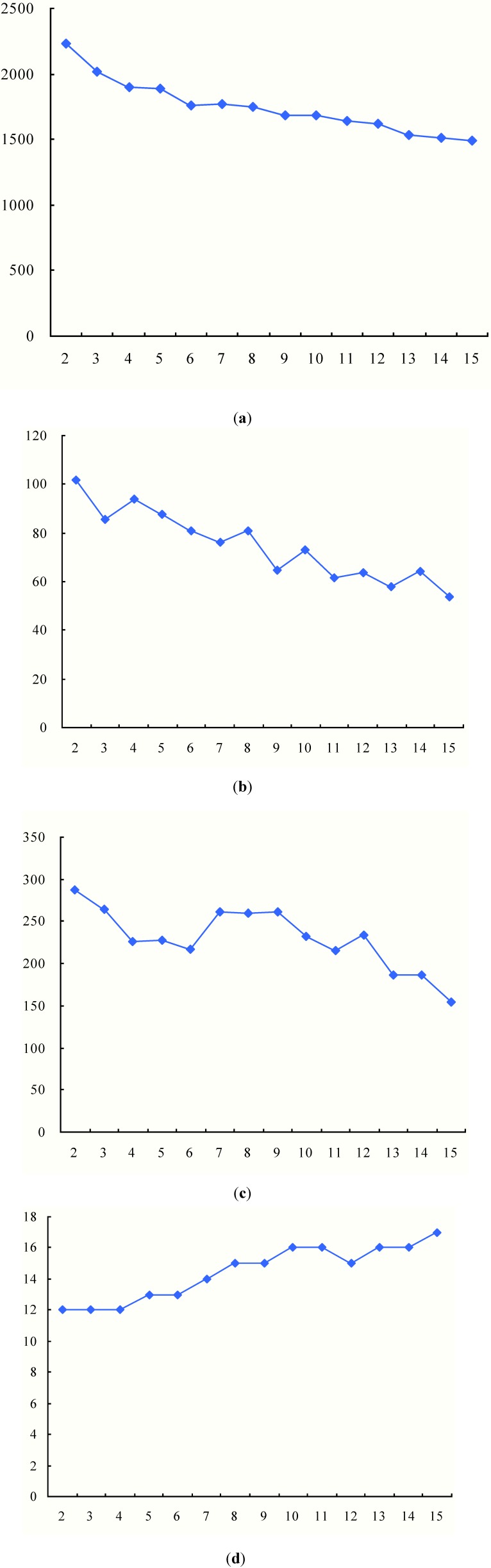
Results with different numbers of TDCs (*Q* = 5000). (**a**) Total duration time; (**b**) Average arrival time; (**c**) Biggest traveling time; (**d**) The number of used vehicles.

In order to show the detailed effects of the number of selected TDCs on the intermodal transportation routes, we compare the results with two and four TDCs. Figure 6a shows the produced delivery routes with two TDCs, and Figure 6b shows the delivery routes with four TDCs (The dotted routes are delivered by helicopters and the solid routes are by vehicles). Table 3 and Figure 7 show the detailed results with different numbers of TDCs.

**Table 3:** Results with different numbers of TDCs (= 5000).

The Number of TDCs	Total Duration Time	Average Arrival Time	Biggest Traveling Time	Number of Used Helicopters	Number of Used Vehicles
2	2236.33	101.69	286.75	2	12
3	2020.92	85.71	264.60	3	12
4	1896.19	93.79	226.22	4	12
5	1884.80	87.89	228.44	5	13
6	1759.45	80.78	217.16	6	13
7	1772.82	75.94	260.95	7	14
8	1746.35	80.62	260.51	8	15
9	1687.84	64.63	261.04	9	15
10	1681.97	73.04	232.67	10	16
11	1643.27	61.73	215.38	11	16
12	1615.44	63.80	233.26	12	15
13	1536.92	57.86	186.36	13	16
14	1512.55	64.16	186.01	14	16
15	1489.22	54.00	154.69	15	17

**Figure 8:**
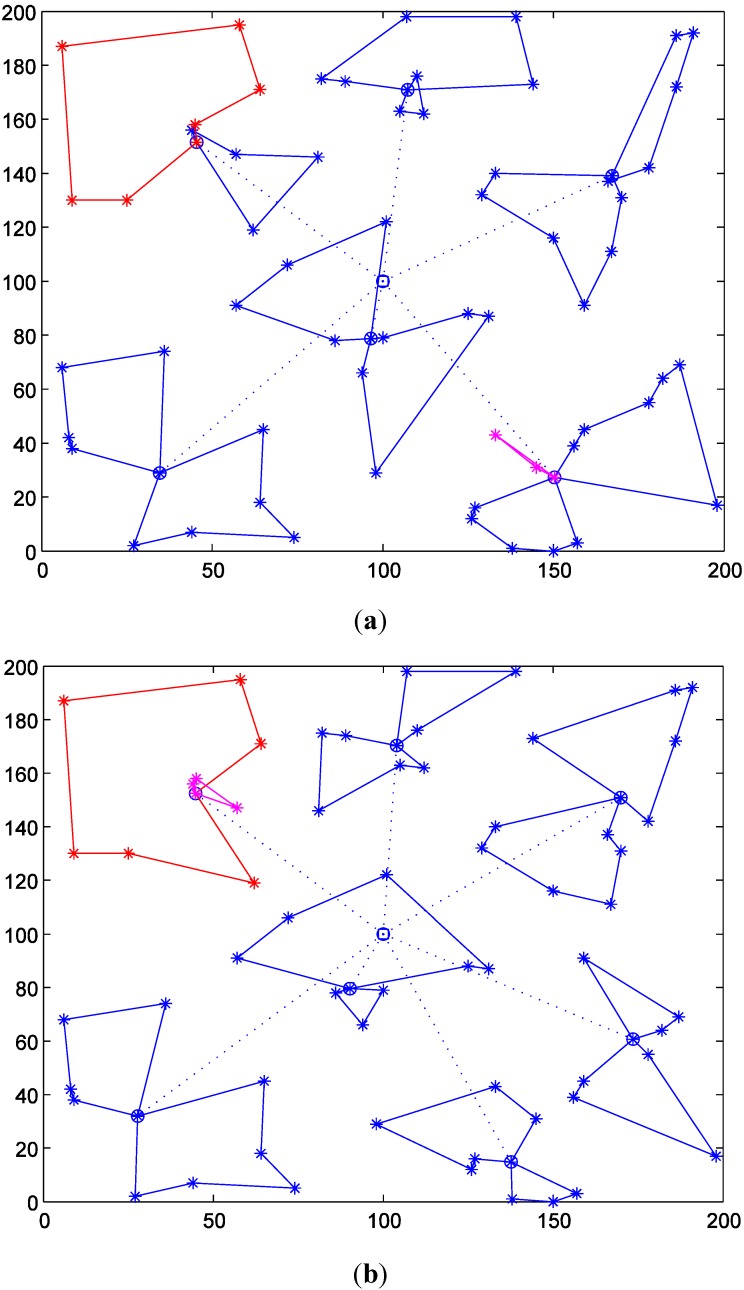
Intermodal transportation routes with six and seven TDCs (*Q* = 5000). (**a**) *m* = 6; (**b**) *m* = 7.

From the results in Table 3 and Figure 7, we can observe the following findings:

(1) With the increase of the number of TDCs, the total duration time would be shortened with a decreasing extent. This is because more TDCs could divide MAPs into smaller groups, which will shorten the vehicle traveling distances, as Figure 6 shows. However, the partitioning effects would be weaker as the groups of MAPs become smaller.

(2) The average arrival time has an approximately fluctuating decrease as the MAPs are assigned to more TDCs. As the number of MAPs covered by TDCs decreases, the arrival time at each MAP tends to be shortened. The fluctuation is due to the uncertainty of the number of MAPs in the partitioning. As analyzed in Section 3.3, the shortened average arrival time will increase the utility of the vaccines.

(3) As shown in Figure 7c, the biggest traveling time is also not absolutely decreased with the increase of the number of TDCs. For example, when the number of TDCs changes from 5 to 12, the biggest traveling time is fluctuating. In order to show the cause of the fluctuation, we compare the delivery routes when the numbers of TDCs are 6 and 7, as Figure 8 shows. The red routes generate the biggest vehicle travel times among all the vehicle delivery routes, which basically determines the biggest travel times of the intermodal transportation due to the contrasting speeds of helicopters and vehicles.

(4) Although the number of total MAPs is not changed, the intermodal transportation with increased TDCs will use more medical vehicles, as Figure 7d shows. Compared with the transportation routes in Figure 6 and Figure 8, we can find that medical vehicles tend to deliver to fewer MAPs because of the decreased number of MAPs covered by more TDCs. Thus, the vehicle capacity may be not used to the largest extent (as the pink routes in Figure 8 show), which certainly increases the total number of used medical vehicles.

#### 4.3.2. Results with Different Vehicle Maximum Capacities

**Figure 9:**
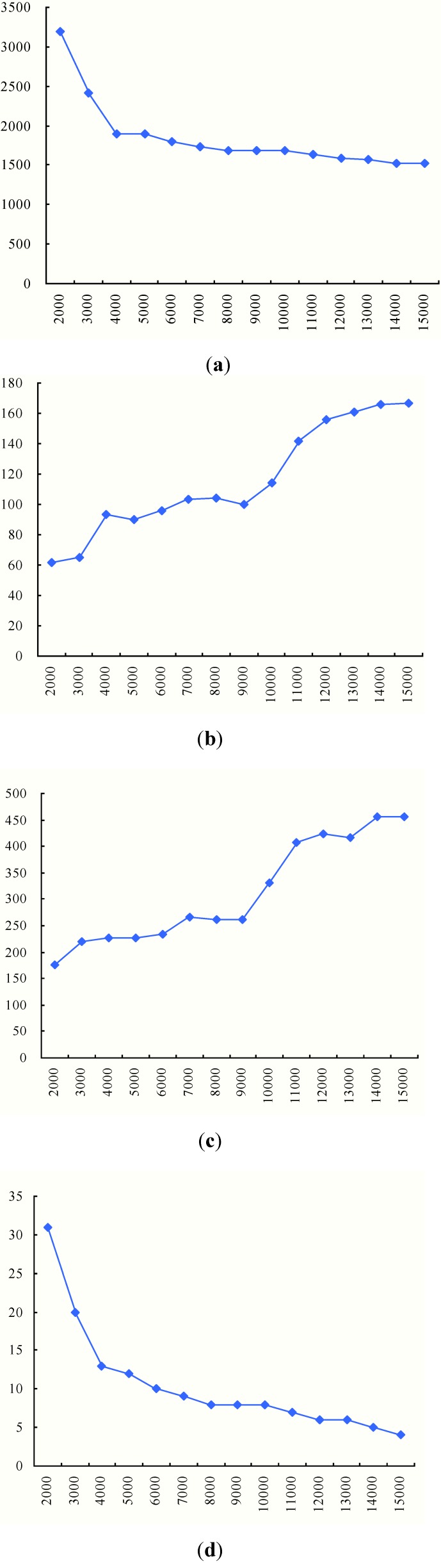
Results with four TDCs and different vehicle maximum capacities. (**a**) Total duration time; (**b**) Average arrival time; (**c**) Biggest traveling time; (**d**) The number of used vehicles.

In above subsection, we set the maximum capacity of medical vehicles as 5000, but the setting may affect the maximum delivery number of MAPs by each medical vehicle. In this subsection, we will investigate the impact of different vehicle maximum capacities on the performance of the intermodal transportation. In order to keep the comparability, we set the number of selected TDCs as 4, and then increase the vehicle maximum capacity from 2000 to 15,000 by 1000. Results are Table 4 and Figure 9 show.

**Table 4:** Results with four TDCs and different vehicle maximum capacities.

Vehicle Maximum Capacity	Total Duration Time	Average Arrival Time	Biggest Traveling Time	Number of Helicopters	Number of Vehicles
2000	3191.10	62.00	175.09	4	31
3000	2406.51	64.96	220.32	4	20
4000	1893.31	93.58	226.89	4	13
5000	1900.70	89.71	226.89	4	12
6000	1798.74	96.05	233.14	4	10
7000	1728.03	103.63	266.03	4	9
8000	1683.76	104.21	261.21	4	8
9000	1684.34	99.59	261.21	4	8
10,000	1679.68	113.86	331.20	4	8
11,000	1636.28	141.39	408.06	4	7
12,000	1584.49	156.00	422.82	4	6
13,000	1565.34	160.76	416.06	4	6
14,000	1529.67	165.57	455.03	4	5
15,000	1515.92	166.57	455.03	4	4

From the results in Table 4 and Figure 9, we can observe the following findings:

(1) As vehicle maximum capacity is enlarged from 2000 to 15,000, the total duration time would decrease. Vehicles with bigger capacities can serve more MAPs, which will decrease the total number of used vehicles. For the given number of MAPs, the decreased in-service vehicles could reduce the total travel distance. However, the decreased extent would become small after the vehicle maximum capacity exceeds 4000, as Figure 9a shows. Especially, the total duration time will not change after the vehicle maximum capacity exceeds 15000, because the medical vehicle with 15,000 doses of vaccines can serve all the MAPs covered by each TDC.

(2) Both average arrival time and biggest traveling time have an increasing trend with the enlargement of vehicle maximum capacity, respectively changing from 62.00 to 166.57 and from 175.09 to 455.03. As analyzed above, the increased traveling distances will also lengthen the average arrival time and biggest traveling time. In details, the two curves increase relatively gently before the vehicle maximum capacity exceeds 9000, but then with sharp increases.

Average arrival time has a direct impact on the availability and utility of medical supplies at MAPs, and biggest traveling time to some extent represents the completion time of the delivery task. Thus, in emergency responses, it is not definitely true that vehicles should load more and more medical supplies. In order to enhance the utility and efficiency of medical supplies, decision makers should make use of medical vehicles with small capacities or not load big vehicles to full capacity if there are available medical vehicles, especially when TDCs cover less MAPs.

#### 4.3.3. Results with Different Helicopter Travel Speeds

**Figure 10:**
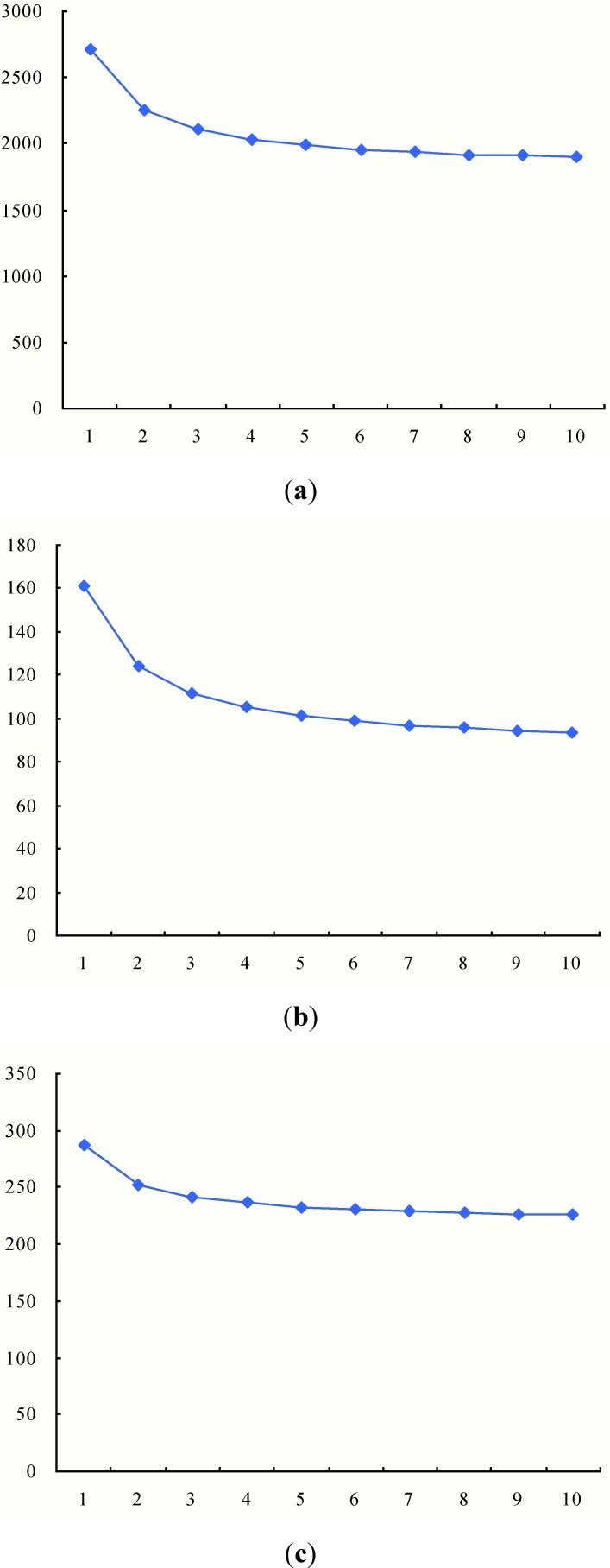
Results with four TDCs and different helicopter travel speeds. (**a**) Total duration time; (**b**) Average arrival time; (**c**) Biggest traveling time.

We further compare the performance of the intermodal transportation with different helicopter traveling speeds. For comparability, we also analyze the results with four TDCs and the vehicle maximum capacity is still set at 5000. Changing the helicopter travel speed from one to 10, the results are as Table 5 and Figure 10 show.

**Table 5:** Results with four TDCs and different helicopter travel speeds.

Helicopter Traveling Speeds	Total Duration Time	Average Arrival Time	Biggest Traveling Time	Number of Helicopters	Number of Vehicles
1	2710.51	161.40	287.49	4	12
2	2258.11	123.84	252.75	4	12
3	2107.31	111.32	241.70	4	12
4	2031.91	105.06	236.17	4	12
5	1986.67	101.30	232.85	4	12
6	1956.51	98.79	230.64	4	12
7	1934.97	97.01	229.06	4	12
8	1918.81	95.66	227.88	4	12
9	1906.24	94.62	226.96	4	12
10	1896.19	93.79	226.22	4	12

The results in Table 5 and Figure 10 indicate that the helicopter traveling speed has an impact on the performance of the intermodal transportation. As the helicopter traveling speed increases from 1 to 10, total duration time, average arrival time and biggest traveling time of the intermodal transportation will decrease, that is, higher helicopter traveling speeds can make full use of the advantage of the intermodal transportation. More accurately, the more contrasting the traveling speeds of helicopters and vehicles are, the more advantageous the intermodal transportation is. However, after the helicopter traveling speed exceeds five times bigger than the vehicle traveling speed, the advantage of the intermodal transportation will decrease to a small extent.

## 5. Conclusions

In developing a solution to a kind of intermodal transportation problem in emergency responses, e have proposed a fuzzy-based method to select the locations of TDCs and assign MAPs to each TDC, and an integer-programming model to determine the optimal delivery routes in the intermodal transportation network. Experimental results verified the effectiveness of the proposed approach, and observed the impacts of the number of selected TDCs, the vehicle maximum capacity and helicopter travel speed on the performance of the intermodal transportation.

There are still further works we will perform. Considerations will be given to reasonably consider the loading and unloading time of medical supplies and reduce the uncertainty of the number of assigned MAPs. Meanwhile, we will improve and apply the approach into real-world disaster responses with more practical considerations such as the geographic situations.

## Appendix I. The Derivation of Equation (7)

By Equation (6), we can get:

*i.e.*,

*i.e.*,

## Appendix II. The Derivation of Equation (8)

By Equation (4), we can get:

*i.e.*,

*i.e.*,

By the above formula, we can get:

*i.e.*,

Substituting Equation (1) into the above formula, we can get:

*i.e.*,

*i.e.*,

Substituting  into the above formula, we can get:

*i.e.*,

*i.e.*,

*i.e.*,
